# Predictive biomarkers of mortality in patients with severe COVID-19 hospitalized in intensive care unit

**DOI:** 10.3389/fimmu.2024.1416715

**Published:** 2024-08-30

**Authors:** Sandrelli Meridiana de Fátima Ramos dos Santos Medeiros, Bruna Maria Nepomuceno Sousa Lino, Vinícius Pietta Perez, Eduardo Sérgio Soares Sousa, Eloiza Helena Campana, Fábio Miyajima, Wlisses Henrique Veloso Carvalho-Silva, Naiara Naiana Dejani, Matheus Santos de Sousa Fernandes, Fatma Hilal Yagin, Fahaid Al-Hashem, Safaa M. Elkholi, Hanan Alyami, Fabrício Oliveira Souto

**Affiliations:** ^1^ Keizo Asami Institute (iLIKA), Federal University of Pernambuco (UFPE), Recife, Pernambuco, Brazil; ^2^ Molecular Biology Laboratory (LABIMOL), Medical Sciences Center, Federal University of Paraíba (CCM/UFPB), João Pessoa, Paraíba, Brazil; ^3^ Fundação Oswaldo Cruz (FIOCRUZ), Eusébio, Ceará, Brazil; ^4^ Department of Physiology and Pathology, Health Sciences Center, Federal University of Paraíba (CCS/UFPB), João Pessoa, Paraíba, Brazil; ^5^ Department of Obstetrics and Gynecology, Medical Sciences Center, Federal University of Paraíba (CCM/UFPB), João Pessoa, Paraíba, Brazil; ^6^ Department of Pharmaceutical Sciences, Health Sciences Center, Federal University of Paraíba (CCS/UFPB), João Pessoa, Paraíba, Brazil; ^7^ Department of Immunology, Instituto Aggeu Magalhães (IAM/FIOCRUZ-PE), Recife, Pernambuco, Brazil; ^8^ Department of Biostatistics and Medical Informatics, Faculty of Medicine, Inonu University, Malatya, Türkiye; ^9^ Department of Physiology, College of Medicine, King Khalid University, Abha, Saudi Arabia; ^10^ Department of Rehabilitation Sciences, College of Health and Rehabilitation Sciences, Princess Nourah bint Abdulrahman University, Riyadh, Saudi Arabia; ^11^ Department of Medical and Surgical Nursing, College of Nursing, Princess Nourah bint Abdulrahman University, Riyadh, Saudi Arabia

**Keywords:** SARS-CoV-2, COVID-19, intensive care unit, critically ill patient, cytokines, chemokines

## Abstract

**Objectives:**

This study was performed to identify predictive markers of worse outcomes in patients with severe COVID-19 in an intensive care unit.

**Methods:**

Sixty patients with severe COVID-19, hospitalized in the Intensive Care Unit (ICU) between March and July 2021, were stratified into two groups according to the outcome survivors and non-survivors. After admission to the ICU, blood samples were collected directly for biomarker analysis. Routine hematological and biochemical biomarkers, as well as serum levels of cytokines, chemokines, and immunoglobulins, were investigated.

**Results:**

Lymphopenia, neutrophilia, and thrombocytopenia were more pronounced in non-surviving patients, while the levels of CRP, AST, creatinine, ferritin, AST, troponin I, urea, magnesium, and potassium were higher in the non-surviving group than the survival group. In addition, serum levels of IL-10, CCL2, CXCL9, and CXCL10 were significantly increased in patients who did not survive. These changes in the biomarkers evaluated were associated with increased mortality in patients with severe COVID-19.

**Conclusion:**

The present study confirmed and expanded the validity of laboratory biomarkers as indicators of mortality in severe COVID-19.

## 1 Introduction

Coronavirus disease 2019 (COVID-19), caused by severe acute respiratory syndrome coronavirus 2 (SARS-CoV-2), has resulted in a severe global public health emergency, causing millions of confirmed cases and associated deaths (1). The clinical features of COVID-19 are heterogeneous, encompassing a broad spectrum of clinical manifestations ranging from mild to moderate and severe forms leading to respiratory, digestive, cardiovascular, renal, or neurological dysfunctions. Patients with a compromised clinical condition due to acute respiratory distress syndrome (ARDS) can develop septic shock, coagulopathies, and even multiple organ failure due to the cytokine storm that leads to respiratory failure and consequently increased mortality (2, 3).

Patients who progress to the most severe form of the disease and require support in the Intensive Care Unit (ICU) have elevated serum levels of pro-inflammatory cytokines, chemokines, and inflammatory mediators, causing multisystem inflammation (4, 5). Several studies have reported an increase in serum levels of cytokines such as interleukin-1 (IL-1), IL-6, IL-2, IL-7, IL-10, IL-12, tumor necrosis factor (TNF-α) and interferon-gamma (IFN-γ) (6–9), which are related to the severity of infection and mortality. Some chemokines were also elevated in patients with severe COVID-19, as chemokine ligand (CCL)-2 (CCL2), CCL3, and chemokine CXC ligands (CXCL), CXCL8, CXCL9, CXCL10, and CXCL11 the most associated with this clinical condition (10, 11).

Similarly, biochemical and hematological markers also show changes during COVID-19 and differ with the severity of the disease. These biomarkers have been used during the evaluation of respiratory impairment and play a vital role in predicting the severity of respiratory distress and guiding the choice of optimal therapy (12, 13). Recent studies have described several biomarkers such as lymphopenia, thrombocytopenia, C-reactive protein (CRP), lactate dehydrogenase (LDH), aspartate aminotransferase (AST), alanine aminotransferase (ALT), d-dimer, ferritin, and troponin associated with worse clinical outcomes and mortality in COVID-19 (14–20).

Despite the diversity of inflammatory mediators related to disease severity, it is still challenging to determine which cytokines and chemokines are strong predictors of progression and mortality due to the heterogeneity with which these mediators present in severe COVID-19 patients (21, 22). In this sense, laboratory findings are a valuable tool for the diagnosis and control of the disease and further studies are needed to better understand which biomarkers can predict the cytokine storm-like syndrome associated with COVID-19 in these patients (3, 13, 23). Thus, this study aimed to identify predictive markers of worse outcomes in severe COVID-19 patients in the intensive care unit, defining the inflammatory profile in these patients.

## 2 Material and methods

### 2.1 Study design and data collection

This is a single-center study, in which we evaluated the profile of patients diagnosed with COVID-19, and admitted to the ICU of a hospital, during the year 2021. The sample was of convenience type. The inclusion criteria were as follows: (1) adult patients (≥18 years) of both sexes; (2) SARS-CoV-2 infection confirmed through reverse transcriptase polymerase chain reaction (RT-qPCR) testing through nasopharyngeal and oropharyngeal swab samples; (3) severe acute respiratory syndrome (SARS); (4) need for ICU admission; (5) Consent was given to participate in the study by close relatives. Exclusion criteria were as follows: (1) age <18 years; (2) pregnancy; (3) inclusion in other clinical studies.

Clinical data, clinical evaluations, and laboratory test results were collected using an electronic system, containing structured patient data. Patients who were discharged from the hospital were designated as survivors, while those who died during hospitalization were designated as non-survivors. This study was approved by the Research Ethics Committee of the Universidade Federal da Paraíba (UFPB) (number 4,026,905).

### 2.2 Viral RNA extraction and RT-qPCR

Nasopharyngeal and oropharyngeal swab samples were collected and stored at -70°C. Viral RNA was extracted using the Maxwell ^®^ Rapid Sample Concentrator 48 (RSC 48) automated extraction system (Promega, USA) and using the Maxwell RSC Viral RNA Extraction Kit, according to the manufacturer’s instructions. SARS-CoV-2 viral RNA was detected by RT-qPCR, using the Allplex™ 2019-nCoV Assay kit (Seegene^®^, Korea), according to the manufacturer’s instructions. A real-time CFX-96 thermal cycler (Bio-Rad Laboratories, Inc., Hercules, CA, USA) was used and the amplification curves were evaluated using the viewer (CFX Manager™ Software-IVD v1.6).

### 2.3 Hematological and biochemical analysis

Blood samples were collected 24-48 h after ICU admission, in Vacutainer^®^ tubes (EDTA, clot activator, and citrate). The following tests were performed: 1) hematological parameters – complete blood count, including an absolute count of neutrophils, lymphocytes, monocytes and platelets, using the Mindray BC-6000 auto hematology analyzer; 2) biochemicals – LDH, ferritin, PCR, AST, ALT, creatinine, urea, troponin I, CK, CK-MB, magnesium, sodium, potassium, calcium, phosphorus, total bilirubin, directly and indirectly. 3) Coagulation markers – D-dimer, fibrinogen, prothrombin time, prothrombin activity time, INR. Hematological and biochemical parameters were compared at different periods of hospitalization, which corresponded to hospital admission, ICU admission, and the clinical outcome (hospital discharge or death).

### 2.4 Determination of anti-Sars-CoV-2 IgG

Blood samples were collected in Vacutainer^®^ tubes with a clot activator after admission to the ICU and processed within 6 hours. Serum was isolated by centrifugation (2500 rpm for 10 minutes) and 500 μl aliquots were stored at -70°C until further analysis of the cytokine profile and antibodies. The SARS-CoV-2 IgG II Quant microparticle chemiluminescent immunoassay (CMIA) was used for the quantitative determination of IgG antibodies to SARS-CoV-2 in Architect and Alinity I systems (Abbott Core Laboratory), following the manufacturer’s instructions. Anti- nucleocapsid protein of SARS-CoV-2 IgG was detected using Qualitative Abbott Architect SARS-CoV-2 IgG assay. The chemiluminescent reaction was measured as a relative light unit (RLU) and expressed as a calculated index (S/C). Semi-quantitative values were calculated from calibrator standards and an index value of 1.4 as the positivity threshold. IgG against S glycoprotein receptor binding domain (anti-S/RBD) was determined using SARS-CoV-2 IgG II Quant kit, a quantitative assay with a cutoff value of 50 AU/mL. Values were converted to Binding Antibodies Units (BAU)/mL to evaluate this antibody titer by the latest notification received from the World Health Organization (WHO) (Notice WHO Standard (20/136) Unit Conversion-RN21040201) (24–27).

### 2.5 Analysis of cytokines and chemokines

Cytokine levels were quantified using the BD™ Cytometric Bead Array (CBA) (BD Biosciences, USA) to detect IL-2, IL-4, IL-6, IL-8, IL-10, IL-17, IFN-γ, TNF-α, CCL2/MCP-1, CCL5/RANTES, CXCL8/IL-8, CXCL9/MIG, and CXCL10/IP-10. However, IL-17 was not detectable, therefore it was excluded from the analysis. Aliquots of the serum samples were thawed, and diluted with assay diluent (1:2 v/v) and CBA analysis was performed according to the manufacturer’s protocol (BD Pharmingen™). The reading was performed using a BD Accuri™ C6 flow cytometer. The analysis was performed using FCAP Array software (BD Biosciences, USA).

### 2.6 Statistical analysis

Statistical analyses were performed using GraphPad Prism software version 9.0.0 (Inc. San Diego, CA, USA) and R software version 4.2.0 (R Core Team). The conformity assessment of the quantitative data was performed using the Shapiro-Wilk test to determine the Gaussian distribution. Data from independent groups were analyzed using the Student’s t-test and Mann-Whitney U test, while data from paired groups were analyzed using the non-parametric Wilcoxon test. Categorical variables were compared using Fisher’s exact test. The median values, as well as the interquartile range (IQR), were used to describe the quantitative data, with the number (n) and percentage (%) representing the categorical variables in the tables. The receiver operating characteristic (ROC) curves were generated in GraphPad Prism, to compare the sensitivity vs. specificity, and the area under those curves (AUC) was used as a measure of test performance. The Kaplan-Meier survival curves were also generated using GraphPad Prism to compare survival time between groups. The variables deemed to have clinical importance and reached p-value ≤0.10 during univariate analyses were included in the logistic regression analysis, and to correct possible confounding factors. The significance level was set at p<0.05 with a 95% confidence interval.

## 3 Results

### 3.1 Clinical characteristics of patients with severe COVID-19

Blood samples were collected from 60 patients with severe COVID-19 24-48 hours after ICU admission. Patients were categorized into survivors and non-survivors based on their clinical outcomes. Patient clinical characteristics and outcomes are shown in **Table 1**. The median age was significantly different between groups, with 59 years (IQR 44-73) for survivors and 69 years (IQR 62-79) for non-survivors (*p*= 0.0209), 51.7% (n=31) of patients were male and 48.3% (n=29) were female. There was no significant difference according to sex between surviving and non-surviving patients (*p*=0.7938). Of these, 80% (n=48) had underlying diseases, including systemic arterial hypertension (SAH) (61.7%, n=37), obesity (45%, n=27), diabetes mellitus (30%, n=18) and cardiovascular diseases (23.3%, n=14). Among the comorbidities, obesity was significantly more prevalent in surviving patients compared to non-surviving patients (*p*= 0.0181), as well as the weight of the surviving patients was significantly higher compared to those who did not survive (*p*= 0.0056).

**Table 1:** Clinical characteristics of severe COVID-19 patients hospitalized in ICU.

Characteristics	All patients (N=60)	CLINICAL OUTCOME		P-value*
		Survivors (N=25)	Non- Survivors (N=35)	
AGE (years), median (IQR)*	67 (51-78)	59 (44-73)	69 (62-79)	0.0209
WEIGHT (kg), mean ± standard deviation**	82 ± 18.3	89.9 ± 15	76.3 ± 11	0.0056
SEX, n (%)				
**Male**	31 (51.7)	12 (48.0)	19 (54.3)	0.7938
**Female**	29 (48.3)	13 (52.0)	16 (45.7)	
COMORBIDITIES, n (%)				
**Systemic arterial hypertension**	37 (61.7)	13 (52.0)	24 (68.5)	0.2817
**Obesity**	27 (45.0)	16 (64.0)	11 (31.4)	0.0181
**Diabetes mellitus**	18 (30.0)	6 (24.0)	12 (34.3)	0.5685
**Cardiovascular disease**	14 (23.3)	3 (12.0)	11 (31.4)	0.1220
**Cancer**	6 (10.0)	2 (8.0)	4 (11.4)	1.000
**Kidney disease**	4 (6.7)	0	4 (11.4)	0.1333
**COPD**	3 (5.0)	1 (4.0)	2 (5.7)	1.000
SYMPTOMS, n (%)				
**Dyspnea**	56 (93.3)	25 (100.0)	31 (88.5)	0.1333
**Cough**	54 (90.0)	22 (88.0)	32 (91.4)	0.6862
**Saturation <95%**	47 (78.3)	21 (84.0)	26 (74.2)	0.5275
**Myalgia**	40 (66.7)	19 (76.0)	21 (60.0)	0.2689
**Fever**	38 (63.3)	14 (56.0)	24 (68.5)	0.4169
**Headache**	28 (46.7)	13 (52.0)	15 (42.8)	0.6014
**Sore throat**	22 (36.7)	5 (20.0)	17 (48.5)	0.0310
**Chills**	21 (35.0)	7 (28.0)	14 (40.0)	0.4157
**Anosmia**	14 (23.3)	9 (36.0)	5 (14.2)	0.0667
**Ageusia**	14 (23.3)	9 (36.0)	5 (14.2)	0.0667
**Diarrhea**	4 (6.7)	3 (12.0)	1 (2.8)	0.2984
DURATION, median (IQR), days				
**Length of hospital stay**	22 (14-29)	23 (16-42)	21 (13-18)	0.0924
**Length of stay in the ICU**	14 (10-23)	13 (11-22)	17 (7-25)	0.9340

*Wilcoxon-Mann-Whitney Test (Shapiro-Wilk: <0,05). **t-Student Test (Shapiro-Wilk: >0,05). IQR, interquartile range; COPD, chronic obstructive pulmonary disease; ICU, intensive care unit.

During treatment, 58.4% (n=35) of the patients did not survive with a median ICU stay of 17 days (IQR 7-25), while 41.6% (n=25) survived and were discharged with a median ICU stay of 13 days (IQR 11-22). A comparison of the patients according to sex was done by plotting the Kaplan Meier curve to assess survival during the hospitalization period; the results presented in **Supplementary Figure 1** did not show a significant difference between the recovery times of female patients compared to male patients.

### 3.2 Vaccination status and antibody response

**Figure 1:**
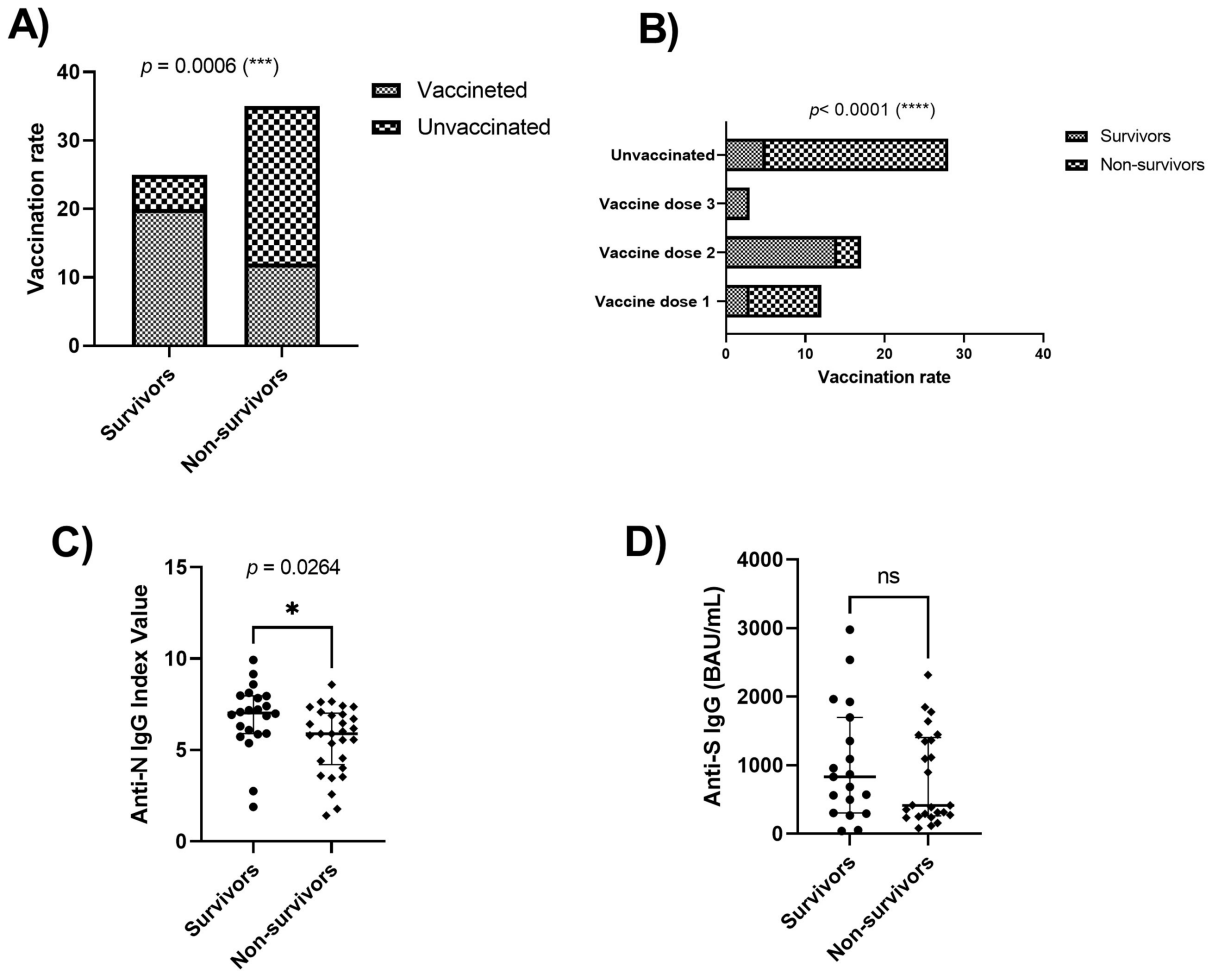
Vaccination rate and IgG levels of patients with severe COVID-19. Severe COVID-19 patients were stratified by survival **(A)** and according to vaccine doses **(B)** (N=60). **(C, D)** anti-N and anti-S protein IgG of SARS-CoV-2 detection in the serum of severe COVID-19 patients (N=58). Mann-Whitney U test. *p=0.0264, (***) p=0.0006, (****) p<0.0001; ns, Non significant.

We evaluated the vaccination status of the patients and the influence on the clinical outcome, and we analyzed the presence of IgG anti-N and IgG anti-S/RBD antibodies in serum samples from severe COVID-19 patients, to determine the antibody response. Overall, the vaccination rate was significantly lower (*p*= 0.0006) in non-surviving patients 34% (12/35), while 80% (20/25) of surviving patients were vaccinated (**Figure 1A**). Many surviving patients presented two doses of the vaccine. In contrast, most of the non-surviving patients were not vaccinated or had received only one dose of vaccine. Patients who had a complete vaccination schedule survived (**Figure 1B**). We also compared the difference in detectability of anti-N and anti-S IgG antibodies between surviving and non-surviving patients. Sample analysis showed that 88% (22/25) of surviving patients were positive for anti-N IgG and anti-S/RBD IgG antibodies, while 83% (29/35) and 85% (30/35) of non-surviving patients were positive for IgG anti-N and IgG anti-S/RBD antibodies, respectively. As shown in (**Figure 1C**), anti-N IgG antibody index values were significantly higher in survivors (*p*= 0.0264), while anti-S/RBD IgG antibody levels (**Figure 1D**) showed no significant difference between groups.

### 3.3 COVID-19 prognostic biomarker levels

**Figure 2:**
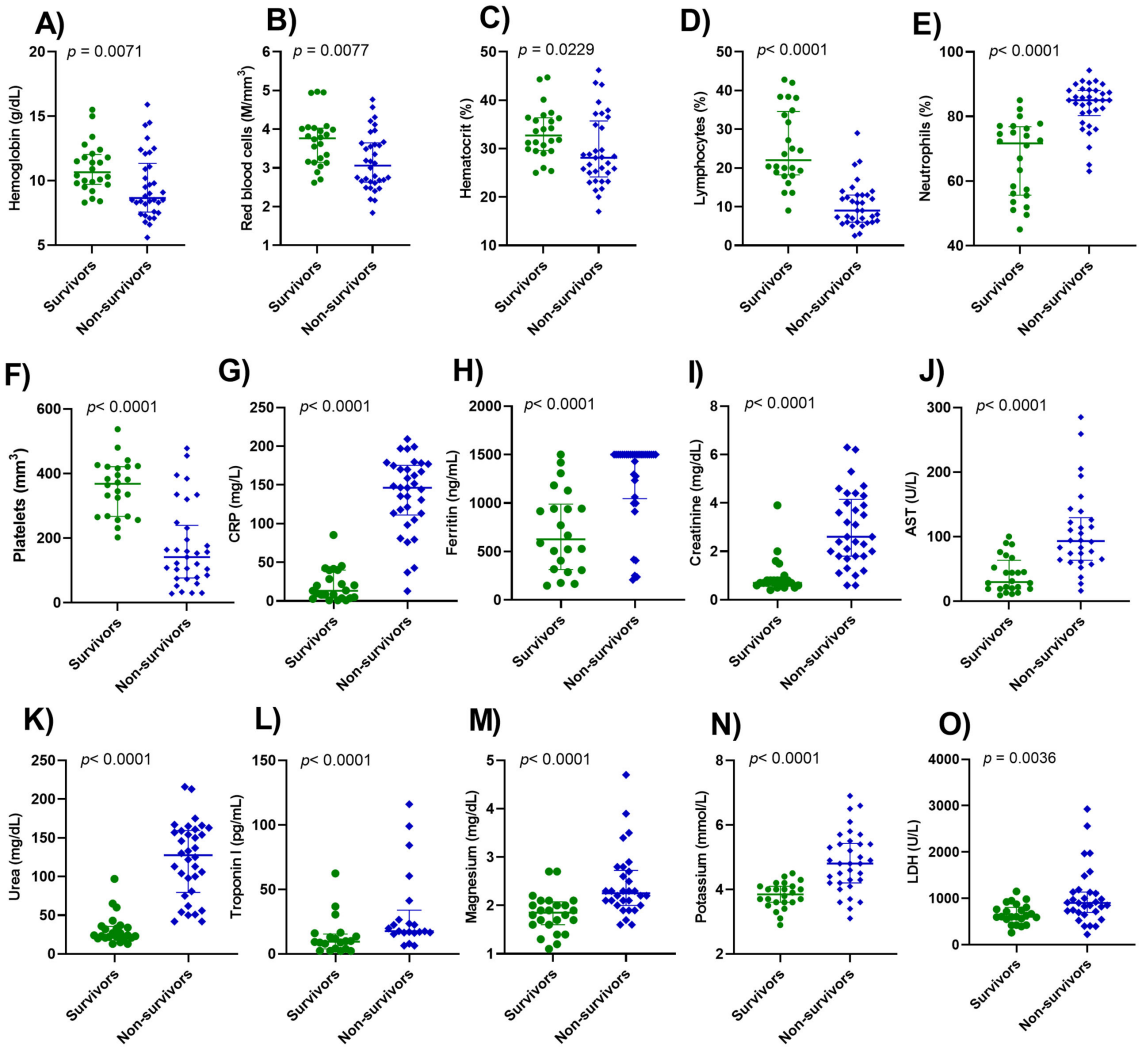
Serum profile of distinct biomarkers in patients with severe COVID-19. The biomarkers analyzed were hemoglobin **(A)**, red blood cells **(B)**, hematocrit **(C)**, lymphocytes **(D)**, neutrophils **(E)**, platelets **(F)**, CRP, C-reactive protein **(G)**, ferritin **(H)**, creatinine **(I)**, AST, aspartate aminotransferase **(J)**, urea **(K)**, troponin I **(L)**, magnesium **(M)**, potassium **(N)**, LDH, lactate dehydrogenase **(O)**. Wilcoxon-Mann-Whitney Test was performed. p< 0.05 was considered statistically significant.

The impact of SARS-CoV-2 infection on hematological parameters showed significant differences between groups. Hemoglobin, red blood cells, and hematocrit levels were significantly lower in non-surviving patients when compared to surviving patients (**Figures 2A–C**). Lymphopenia and neutrophilia were more pronounced in non-surviving patients (**Figures 2D, E**) while reduced numbers of platelets were observed in non-surviving patients (**Figure 2F**). Levels of CRP, ferritin, creatinine, AST, and urea, troponin I, magnesium, potassium, and LDH were significantly higher in non-surviving patients compared with those who survived (**Figures 2G–O**).

Several inflammatory mediators are involved in the immunopathology of COVID-19. We investigated some cytokines and chemokines in the serum of patients with severe COVID-19 admitted to ICU. Blood samples were collected 24-28 hours after hospitalization, and the levels of cytokines and chemokines were correlated with clinical outcome, discharge (survivors), or death (non-survivors). As shown in **Table 2**, non-surviving patients had higher levels of IL-10 than survivor patients (*p*<0.0001). The levels of cytokines IL-2, IL-4, IL-6, IL-8, IFN-γ, and TNF-α were not significantly different between groups. The levels of CCL2, CXCL9, and CXCL10 were significantly higher in non-surviving than surviving patients with (*p*=0.0002), (*p*=0.0023), and (*p*=0.0043) respectively, while CCL5 was significantly lower in non-surviving than surviving patients (*p*=0.0070).

**Table 2:** Cytokines and chemokines levels in the serum of severe COVID-19 patients.

Cytokines (pg/mL)	CLINICAL OUTCOME, median (IQR)		P-value*
	Survivors (N=25)	Non- survivors (N=35)	
**IFN-γ**	62.5 (60.2-65.0)	62.7 (61.4-65.2)	0.5772
**TNF-α**	49.2 (46.5-51.9)	49.2 (46.8-51.7)	0.8827
**IL-2**	75.4 (73.9-76.5)	76.0 (74.9-76.7)	0.1608
**IL-4**	59.9 (59.0-61.5)	60.5 (58.6-61.6)	0.7929
**IL-6**	71.5 (66.4-79.2)	79.9 (67.1-114.0)	0.3094
**IL-8**	1049.4 (734.3-1381.2)	1231.5 (728.9-3027.0)	0.1999
**IL-10**	77.5 (71.5-88.9)	114.2 (99.3-173.6)	<0.0001
**CCL2**	453.1 (302.4-1205.7)	3238.3 (993.3-6024.7)	0.0002
**CCL5**	111001 (99535-125250)	94120 (76783-110068)	0.0070
**CXCL9**	2175.9 (1022.2-4805.2)	5271.8 (2551.4-14971.9)	0.0023
**CXCL10**	644.0 (487.1-1687.1)	2591.2 (862.5-5062.5)	0.0043

*Wilcoxon-Mann-Whitney Test (Shapiro-Wilk: <0,05).

IQR, interquartile range.

**Figure 3:**
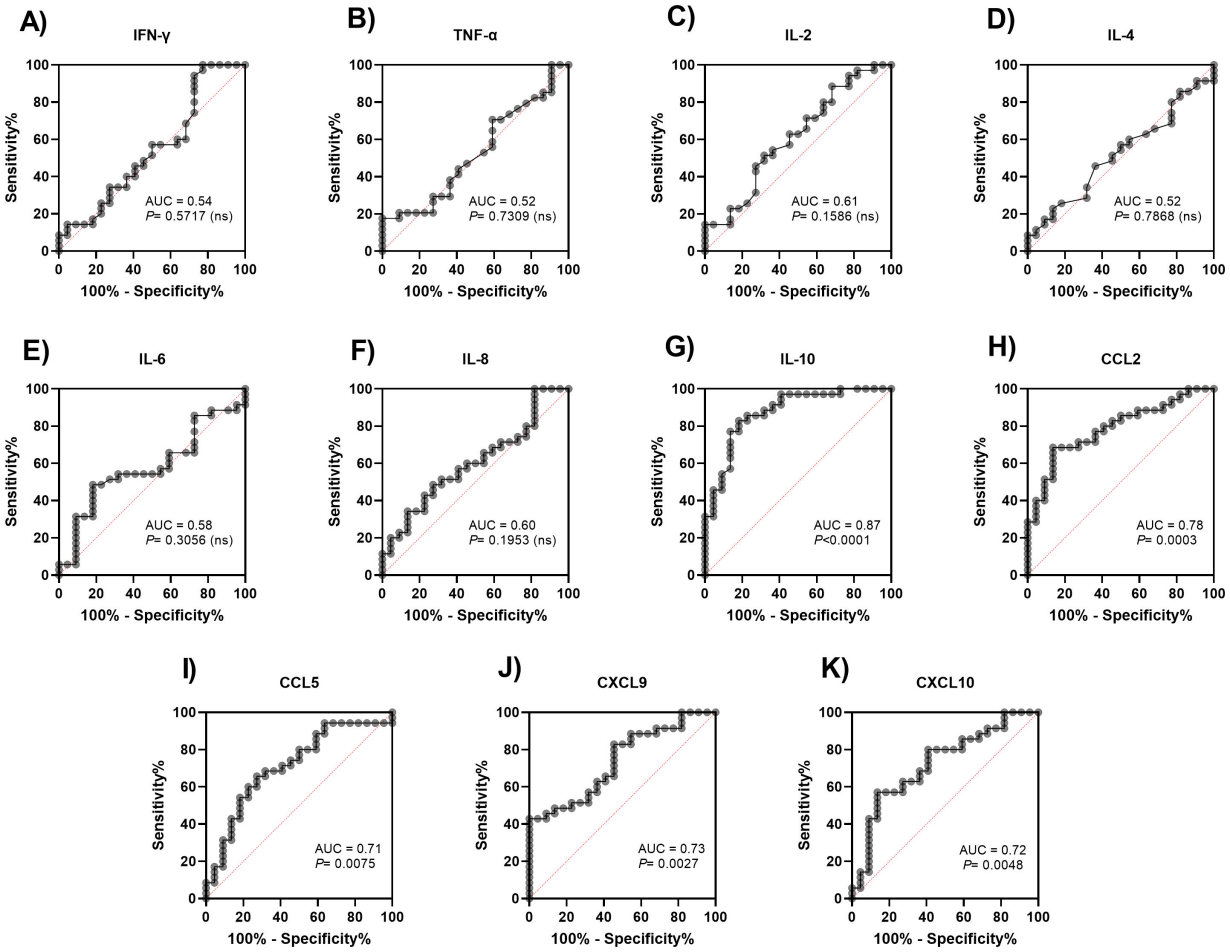
Receiver operating characteristic (ROC) curve of cytokines and chemokines to predict mortality in severe COVID-19 patients. Patients with COVID-19 were stratified into survivors and non-survivors. The ROC curve analysis was performed for IL-2 **(A)**, IL-4 **(B)**, IL-6 **(C)**, IL-8 **(D)**, IL-10 **(E)**, TNF-α **(F)**, IFN-γ **(G)**, CCL2 **(H)**, CCL5 **(I)**, CXCL9 **(J)**, CXCL10 **(K)**.

**Figure 4:**
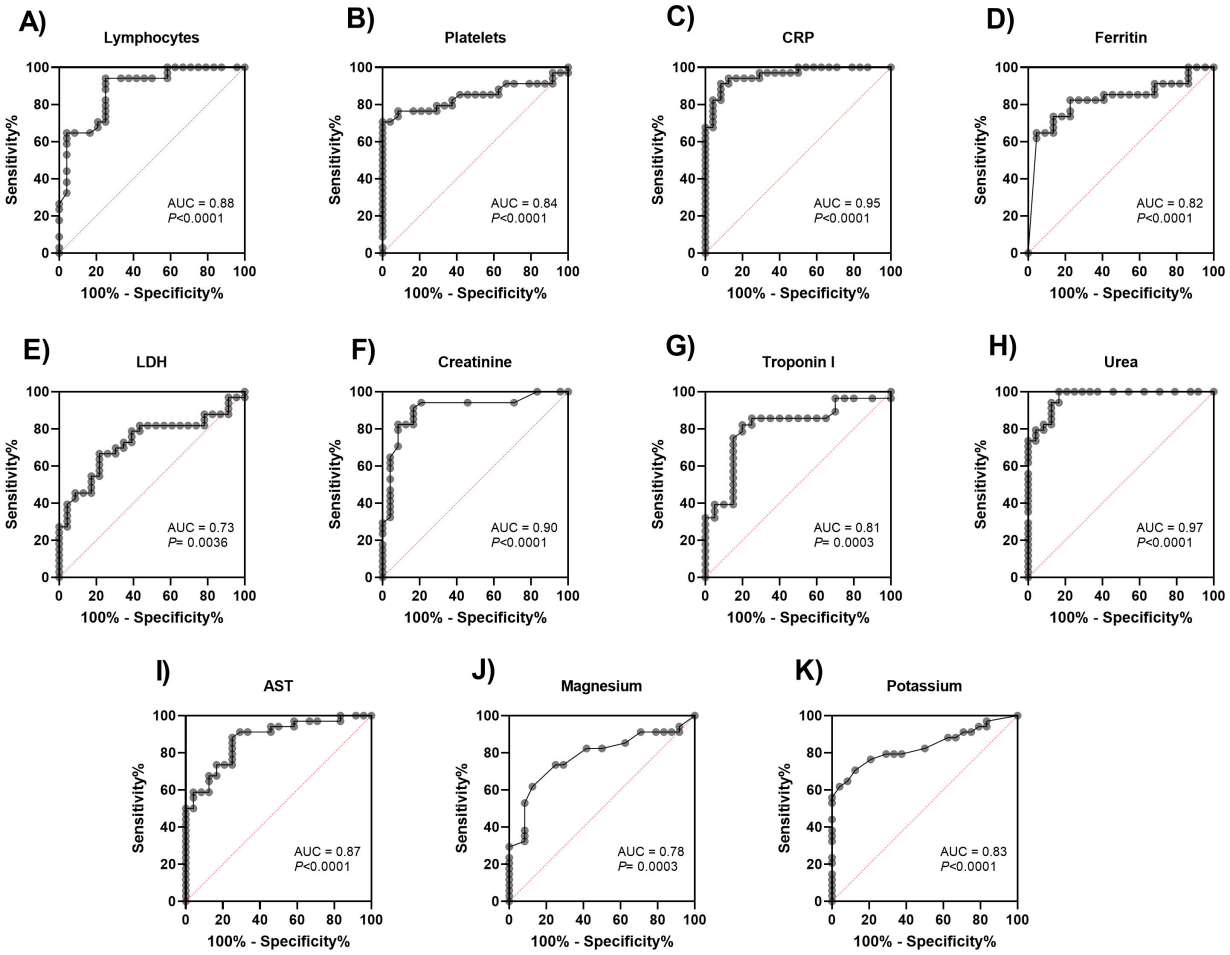
Receiver operating characteristic (ROC) curve of distinct biomarkers for relevance in predicting mortality in patients with severe COVID-19. The biomarkers were analyzed in blood samples from 60 patients. **(A)** Lymphocytes, **(B)** Platelets, **(C)** CRP, C-reactive protein, **(D)** Ferritin, **(E)** LDH, **(F)** Creatinine, **(G)** Troponin I, **(H)** Urea, **(I)** AST, aspartate aminotransferase, **(J)** Magnesium and **(K)** Potassium.

**Figure 3** shows the Receiver Operating Characteristic (ROC) curve comparing the serum levels of cytokines and chemokines with the predicted probability of mortality of the patients evaluated. The areas under the curve (AUC) revealed the high performance of IL-10 (AUC: 0.874; 95% CI 0.780-0.969), while the chemokines CCL2, CCL5, CXCL9 and CXCL10 revealed the adequate performance in predicting the mortality of patients evaluated (AUC: 0.785; 95% CI 0.667-0.903), (AUC: 0.711; 95% CI 0.572-0.851), (AUC: 0.737; 95% CI 0.609-0.865) and (AUC: 0.723; 95% CI 0.586-0.860), respectively. Other biomarkers were also evaluated for their accuracy in predicting mortality in patients with severe COVID-19. The results are described in **Figure 4**. All information on the AUCs of the biomarkers evaluated is presented in **Supplementary Table 1**.

### 3.4 Multivariate logistic regression analysis

A logistic regression model was performed with clinical (age and weight) and immunological (IL-10, CCL2, CCL5, CXCL9, and CXCL10) variables previously associated with clinical outcome to correct confounding factors such as those related to age and weight. The results maintained a significant association of elevated IL-10 (*p*=0.0082) and CCL2 (*p*=0.0267) levels as risk factors (**Table 3**), being significantly higher in non-survivors. The model’s internal validation showed that the analysis was accurate and had high adherence (AUROC=0.8377; Z=1.1649; *p*=0.2441) with the concordance of 95.2%, thus being appropriate for predicting outcomes in severe COVID-19 patients.

**Table 3:** Variables included in a fitted logistic regression model to explain clinical outcome of severe COVID-19 patients.

Variables	Estimate (β)*	OR	95% CI	*P*
Weight (kg)	-0.016370	0.9838	0.9217 – 1.0501	0.623
Age (years)	-0.006394	0.9936	0.9250 – 1.0673	0.861
IL10 (pg/mL)	0.071650	1.0743	1.0187 – 1.1329	0.008
CCL2 (pg/mL)	0.007354	1.0074	1.0067 – 1.0080	0.027
CCL5 (pg/mL)	-0.000014	1.0000	1.0000 – 1.0000	0.434
CXCL9 (pg/mL)	0.000186	1.0002	1.0000 – 1.0004	0.116
CXC10 (pg/mL)	-0.000236	0.9998	0.9995 – 1.0000	0.072
(intercept)	-3.811000	_	_	0.4732

CI, confidence interval; OR, odds ratio.

*Model’s internal validation: AUROC=0.8377; Z=1.1649; P=0.2441.

## 4 Discussion

Mortality in COVID-19 patients has been associated with a hyperinflammatory immune response, characterized by cytokine storm and multiple organ failure (28). In addition to cytokines, hematological and biochemical biomarkers also present alterations that are fundamental in the classification of the severity and prognosis of COVID-19 (29). In this study, lymphopenia, neutrophilia, anemia, and thrombocytopenia were the key features of non-survivors with COVID-19. The levels of CRP, AST, creatinine, ferritin, AST, troponin I, urea, magnesium, and potassium were also enhanced in non-survivors. In addition, elevated levels of IL-10, CCL2, CXCL9, and CXCL10; and reduced levels of CCL5 were observed in non-survivors. These changes in the evaluated biomarkers were associated with increased mortality in patients with severe COVID-19.

The analysis of the clinical profile between the groups shows that patients who did not survive were older than those who survived. Some immunological mechanisms responsible for the increased risk of death from COVID-19 in the elderly are raised. Immunosenescence, which causes age-related changes in innate and adaptive immunity, has been associated with increased mortality in older adults infected with SARS-CoV-2 (30). The elderly have a deficient immune response to SARS-CoV-2 infection, due to progressive biological changes in the immune system that lead to a decline in its functions. These alterations, associated with comorbidities, cause greater vulnerability in older individuals to developing infections, increasing COVID-19 morbidity and mortality (31, 32). In addition, sex does not seem to influence the survival of the patients in the study since no significant differences were found between surviving patients and those who did not survive. There were also no significant differences between the recovery times of female patients compared to male patients.

In this study, obesity was more prevalent among patients who survived the infection. Studies affirm a possible obesity paradox, as this comorbidity is a significantly higher risk factor for severity and ICU admission, but it is not associated with an increased risk of death (33–35). Some explanations have been proposed to describe this inverse association between obesity and mortality risk. Among them, the secretion of immunomodulatory substances by fat cells such as leptin, IL-10, and soluble TNF-α receptor, can attenuate an inflammatory response and increase metabolic reserve due to excess fat reserves, can counteract the increase in catabolic stress and improve the survival of patients during critical illnesses (36). Another factor that can also influence the prevalence of obesity is the geographic region and the population studied (37).

In the humoral immune response against SARS-CoV-2, B cells stimulate the immune response against the N protein in the early phase of acute disease, while antibodies against the S protein can be identified between 4 to 8 days after the onset of symptoms (38, 39) The significant difference between the anti-N IgG antibody index values demonstrates that this phase of response against SARS-CoV-2 infection was stronger in surviving patients compared to non-survivors. As previously reported, we did not observe significant differences in anti-S/RBD IgG titers between surviving and non-surviving patients, showing that the combination of immunity provided by the infection and vaccines did not increase the production of specific antibodies in the patients evaluated. However, the high positivity rate of severe COVID-19 patients for anti-N and anti-S/RBD antibodies, surpassing the vaccination rate, shows that the response of these antibodies is related to the severity of COVID-19 (40).

The pathophysiology of severe infection is marked by elevated neutrophils and reduced lymphocyte counts in the blood (12, 41, 42). The immune response marked by severe lymphopenia increases the chance of late complications and early expression of proinflammatory cytokines during lung injury caused by SARS-CoV-2, while neutrophilia occurs mainly in patients with severe COVID-19 because of the inflammatory state caused by cytokine storm. However, extensive neutrophil infiltration into the lungs is a key point in the acute inflammatory response to eradicate pathogens (43).

Anemia and thrombocytopenia have been described in patients with severe COVID-19 (44), and corroborating our findings, Jha et al. (17) showed a significant correlation between anemia and death in patients with severe COVID-19, which suggests anemia is an important parameter in predicting disease mortality. Non-surviving patients also showed changes in CRP, ferritin, creatinine, AST, LDH, urea, troponin I, magnesium, and potassium levels. Most of the patients in our study were over 60 years old. CRP was associated with severe COVID-19 and is also considered a predictor of in-hospital mortality in the elderly (16).

The level of circulating ferritin increases during viral infections, being a biomarker of immune dysregulation, especially under extreme hyperferritinemia, through direct immunosuppressive and pro-inflammatory effects (45). We also observed elevated levels of ferritin, creatinine, and ALT in non-survivors. During the cytokine storm in COVID-19, pro-inflammatory cytokines may stimulate hepatocytes, Kupffer cells, and macrophages to secrete ferritin (46–48). The acute decline in renal function is a frequent complication of COVID-19 and creatinine levels are a hallmark of requiring ICU treatment (49). Hepatic biochemical abnormalities, liver damage, and ALT level enhancement have been expected during severe COVID-19 infection due to the direct and indirect impact of SARS-CoV-2 in the liver (14, 50).

Elevated levels of inflammatory mediators have been associated with worse prognosis during COVID-19 (3, 22, 51). IL-6 is a pro-inflammatory cytokine primarily produced during acute and chronic inflammation by macrophages and activated T cells during viral infection (52). Additionally, IL-6 is produced by lung epithelial cells in response to stimuli such as allergens and respiratory viruses (28). In COVID-19, IL-6 plays a significant role in cytokine storms. In this scenario, IL-6 acts on the endothelial cells of the pulmonary capillaries, leading in the most severe cases of COVID-19, to an excessive and uncontrolled immune response (53, 54). Although studies have related IL-6 as a biomarker of progression and severity in COVID-19, in our study no difference was observed in serum IL-6 levels between the groups evaluated.

In turn, IL-10 is an anti-inflammatory cytokine that exerts immunosuppressive effects on innate and adaptive inflammation (28). Patients with severe COVID-19 who did not survive the disease have higher levels of IL-10 compared to surviving patients (55). Studies suggest that increased IL-10 may exacerbate the pathogenesis involved in COVID-19 severity, thus elevated IL-10 expression is considered an indicator of poor prognosis in COVID-19 (28, 53, 56). These findings corroborate our findings in which serum levels of IL-10 were significantly higher in non-survivors than survivors.

Lu et al. (57) suggest that chemokines such as CCL2/MCP-1, CCL5/RANTES, and CXCL10/IP-10 initiate the deadly immunopathological pathway of COVID-19. CCL2, also known as monocyte chemoattractant protein-1 (MCP-1), acts as a regulator of the migration and infiltration of monocytes and macrophages during inflammatory response in various infectious processes (58). In association with other inflammatory cytokines, the increase in CCL2/MCP-1 in COVID-19 leads to harmful disease progression and induces acute kidney injury in critically ill patients (59). In addition, CCL2/MCP-1 levels are shown to be upregulated during the early phase of infection and increase significantly during the late stages of the disease in non-surviving patients (60). In our study, CCL2/MCP-1 showed a significant increase in non-surviving patients compared to surviving patients.

CCL5/RANTES is a leukocyte chemoattractant that binds to CCR1, CCR3, and CCR5-like receptors. We observed a reduction in CCL5 levels in non-surviving patients compared to those who survived. Teixeira et al. (61), describe higher levels of CCL5/RANTES in patients who survived COVID-19 compared to those who did not survive. Overall, studies have shown that CCL5/RANTES levels increased significantly in patients with mild disease compared to critically ill patients in the ICU. In addition, it has been reported that in the early phase of SARS-CoV-2 infection, serum CCL5 levels are increased in patients with mild symptoms of COVID-19 compared to severe patients (62–64). These reports suggest that CCL5/RANTES may protect against viral infection before lung inflammation and disease progression occur. Thus, CCL5/RANTES is an important biomarker in antiviral responses and recovery in patients with mild COVID-19 (62, 65).

CXCL9/MIG and CXCL10/IP-10 are members of the CXC chemokine family and are often referred to as IFN-inducible CXCR3 chemokines. These chemokines share IFNγ as the primary inducer and CXCR3 as the G protein-coupled receptor (66). We observed that CXCL9/MIG levels were significantly increased in non-survivor patients compared to survivors. Patients with severe COVID-19 showed higher serum levels of CXCL9/MIG than those with mild to moderate disease (67). CXCL10, also known as interferon-induced protein 10 (IP-10), is a chemoattractant for monocytes/macrophages, dendritic cells, natural killer cells, and T cells. Here, CXCL10/IP-10 showed a significant increase in non-surviving patients compared to those who survived the disease, corroborating to (10) study, which shows that increased CXCL10/IP-10 expression is correlated with severe acute respiratory syndrome due COVID-19 (10) and corroborating the study by Laudanski et al. (68) who identified higher serum CXCL10 levels in non-survivor patients compared to the survival group.

The main limitation of this study was that we assessed the levels of inflammatory cytokines and chemokines only at ICU admission, but we were unable to obtain repeated collections or evaluations during follow-up. Thus, future studies should be conducted with continuous measurements of these inflammatory mediators in COVID-19 infection to assess serum levels throughout the ICU hospitalization period. Furthermore, we evaluated hospitalized patients only during the year 2021. Additional studies that can focus on the profile of patients in different periods are needed.

## 5 Conclusions

This study shows changes in hematological, biochemical, and inflammatory parameters in COVID-19 non-surviving patients. Lymphopenia, neutrophilia, and thrombocytopenia, as well as increased levels of CRP, AST, creatinine, ferritin, AST, troponin I, urea, magnesium, and potassium served as prognostic markers and were associated with patient mortality. Changes in IL-10, CCL2, CXCL9, and CXCL10 serum levels were also associated with increased mortality in patients. Thus, this study confirms the relationship between the various biomarkers evaluated in hospitalized patients with severe COVID-19 and their relationship with fatal COVID-19. The results corroborate previous studies conducted in other countries and reinforce the importance of identifying potential targets that can reduce the expression of these biomarkers as a strategy in treating and controlling viral respiratory infections.

## Data Availability

The original contributions presented in the study are included in the article/**Supplementary Material**. Further inquiries can be directed to the corresponding authors.
