# Supplementary material for: Predictive biomarkers of mortality in patients with severe COVID-19 hospitalized in intensive care unit
Source: Front Immunol. 2024 Aug 30;15:1416715. doi: 10.3389/fimmu.2024.1416715 (PMC11401048; doi:10.3389/fimmu.2024.1416715)
Supplement: Supplementary file 1 [file DataSheet1.docx]

***Supplementary Material***

# Supplementary Figure 1


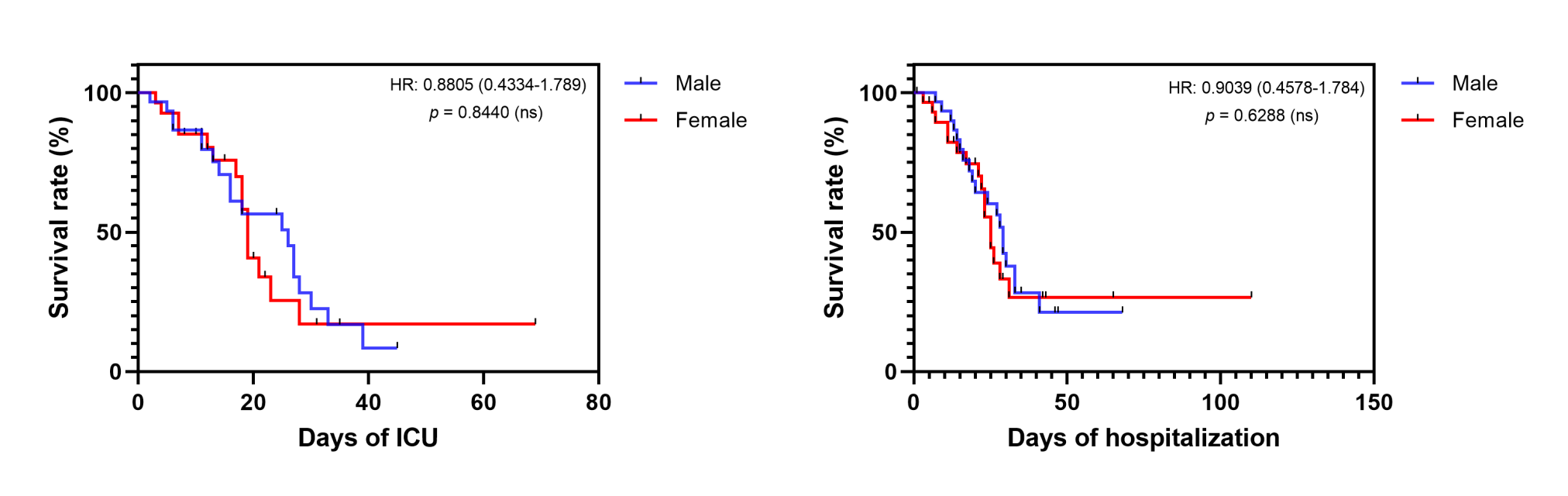


**Supplementary Figure 1.** Kaplan-Meier survival curves of patients with severe COVID-19, according to **(A)** length of ICU stay and **(B)** total length of hospital stay. ns: not significant.

# Supplementary Table 1. Areas under the curve analysis: the biomarkers’ capacity in predicting mortality.

| Area under the curve | | | | | |
| --- | --- | --- | --- | --- | --- |
| Test Result Variables | **Area** | **Standard Error** | **P-value** | **95% confidence interval** | |
|  |  |  |  | **Lower Bound** | **Upper Bound** |
| Lymphocytes | 0.880 | 0.045 | <0.0001 | 0.791 | 0.969 |
| Platelets | 0.843 | 0.054 | <0.0001 | 0.737 | 0.949 |
| C-reactive protein | 0.959 | 0.022 | <0.0001 | 0.914 | 1.000 |
| Ferritin | 0.824 | 0.057 | <0.0001 | 0.712 | 0.936 |
| LDH | 0.730 | 0.067 | 0.0036 | 0.598 | 0.863 |
| Creatinine | 0.906 | 0.042 | <0.0001 | 0.823 | 0.990 |
| Troponin I | 0.810 | 0.065 | 0.0003 | 0.682 | 0.938 |
| Urea | 0.970 | 0.018 | <0.0001 | 0.935 | 1.000 |
| AST | 0.875 | 0.044 | <0.0001 | 0.787 | 0.963 |
| Magnesium | 0.780 | 0.061 | 0.0003 | 0.659 | 0.900 |
| Potassium | 0.833 | 0.053 | <0.0001 | 0.729 | 0.937 |
| IL-10 | 0.874 | 0.048 | <0.0001 | 0.780 | 0.969 |
| CCL2 | 0.785 | 0.060 | 0.0003 | 0.667 | 0.903 |
| CCL5 | 0.711 | 0.071 | 0.0075 | 0.572 | 0.851 |
| CXCL9 | 0.737 | 0.065 | 0.0027 | 0.609 | 0.865 |
| CXCL10 | 0.723 | 0.069 | 0.0048 | 0.586 | 0.860 |
